# Synchronous Responses of Plant Functional Traits to Nitrogen Deposition From Dominant Species to Functional Groups and Whole Communities in Alpine Grasslands on the Qinghai-Tibetan Plateau

**DOI:** 10.3389/fpls.2022.827035

**Published:** 2022-02-09

**Authors:** Shuai Li, Zhenzhen Zhao, Shikui Dong, Hao Shen, Yudan Xu, Jiannan Xiao, Xiaoxia Gao, Shengnan Wu, Paul Stufkens

**Affiliations:** ^1^College of Resource and Environment, Shanxi Agricultural University, Jinzhong, China; ^2^School of Environmental and Chemical Engineering, Shanghai University, Shanghai, China; ^3^School of Grassland Science, Beijing Forestry University, Beijing, China; ^4^College of Grassland Science, Shanxi Agricultural University, Jinzhong, China; ^5^School of Environment, Beijing Normal University, Beijing, China; ^6^English Department, Zamorano University, Tegucigalpa, Honduras

**Keywords:** plant functional traits, nitrogen deposition, alpine steppe, cultivated grassland, Qinghai-Tibet Plateau

## Abstract

Nitrogen deposition is recognized as one of the major threats to the ecosystem function of alpine grasslands on the Qinghai-Tibetan Plateau (QTP). However, few studies have documented the gradient responses of plant species, functional groups, and communities in alpine grassland ecosystems to various levels of N deposition on the QTP. We applied eight linear mixed-effect models combing acidification, eutrophication, and phosphorus availability to explore if the responses of functional traits (particularly plant height and specific leaf area) of plants from dominant species to functional groups and whole communities in different types of grassland to nitrogen deposition were consistent with the same or different models. We found that the specific leaf area of *Stipa capillata*, non-forb, and community-weighted mean value in the alpine steppe were synchronous and related to acidification with nitrogen addition; the height of *Stipa capillata*, non-forb, and community-weighted mean value in the alpine steppe was synchronous and related to acidification, eutrophication, and phosphorus availability with nitrogen addition; the height and specific leaf area of *Elymus breviaristatus* to functional groups and community-weighted mean value in cultivated grasslands (CGs) were synchronous and related to acidification, eutrophication, and phosphorus availability with nitrogen addition. Most of the responses of functional traits of plants to acidification, eutrophication, and phosphorus availability associated with nitrogen deposition in the alpine steppe and the CG were synchronous, while only the response of the specific leaf area of forb functional groups to eutrophication associated with N deposition in the alpine steppe was asynchronous.

## Highlights

– Dominant specie, non-forb, and community were related to acidification with N addition in AS.
– Responses of height were more sensitive to N addition than SLA in both AS and CG.
– Responses of SLA and height in CG were more sensitive to N addition than AS.
– Responses of SLA of dominant specie, non-forb, and community were synchronous with N addition.
– Only response of SLA of forb was asynchronous compared to others with N addition in AS.

## Introduction

Nitrogen (N) deposition is recognized as one of the major threats to global biodiversity (Phoenix et al., 2010; Payne et al., 2017), which has increased sixfold since the 1860s (Field et al., 2014) and is expected to continue to increase in the future on a global scale (Bobbink et al., 2010). In China, the average N deposition rate was 13.2 kg N/ha/year in the 1980s and became 21.1 kg N/ha/year in the 2000s (Liu et al., 2013). On the Qinghai-Tibetan Plateau (QTP), known as “the Third Pole of the Earth,” a significant increase in the N deposition rate has been found since the 1980s (Chen et al., 2013; Liu et al., 2013), and the current rate ranges from 8.7 to 13.8 kg N/ha/year in the 2000s (Liu et al., 2013). Alpine grasslands, including mainly alpine meadow, alpine steppe (AS), and alpine desert steppe, occupy more than half the area of the QTP territory (Dong et al., 2010; Zhao et al., 2017), with a low-atmospheric N-input environment historically (Zhang et al., 2014), and are very sensitive to N deposition (Piao et al., 2011; Shen et al., 2014; Ma et al., 2019). However, few studies have documented the gradient responses of plant species, functional groups, and communities in alpine grassland ecosystems to various levels of N deposition on the QTP.

It is widely reported that there are mainly three pathways in which N deposition can affect a plant community, namely, acidification, eutrophication, and phosphorus (P) limit (Phoenix et al., 2010, 2012; Stevens et al., 2010). Soil acidity derived directly or indirectly from N deposition (Liu et al., 2012; Greaver et al., 2016) can lead to migration of metals, a loss of basic cations, an imbalance among N-containing compounds, and the ultimate alteration of THE composition of a plant community (Stevens et al., 2010; Laliberté et al., 2014). N is generally known to be the limiting factor in most terrestrial ecosystems (Jing et al., 2016; Wang et al., 2018). Some scholars believe that an increasing N deposition rate may lead to the transition from N limitation to N saturation in ecosystems (Lu et al., 2010), which may benefit the coexistence of species by excluding non-nitrophilous plants (Bobbink et al., 2010). In contrast, some other scholars think that the immobilization of NO_3_-N was reduced and NH_4_-N was increased in the process of transition from N limitation to N saturation (Phoenix et al., 2012), which may limit the coexistence of species due to differences in plant preferences and tolerances of NH_4_-N or NO_3_-N (van den Berg et al., 2016). Some studies have shown that other nutrient limitations (such as P) become more important in the context of an increased rate of N deposition, although N is still the prime limiting factor in most ecosystems (Lu et al., 2010; Stevens et al., 2010; Phoenix et al., 2012), since the main driver for activating P in the soil is weathering (Cleland and Harpole, 2010). Particularly, in a low-temperature environment, such as alpine grassland areas of the QTP, the uptake of P by plants will be limited (Laliberté et al., 2014). However, these three pathways are not necessarily independent, as the absorption of N and P by plants is synergistic (Stevens et al., 2010; Phoenix et al., 2012), and the pH also has an effect on the absorption of N and P in plants (Laliberté et al., 2014).

The response of plant communities to N deposition is mostly focused on plant richness in the field of academic studies (Zavaleta et al., 2003; Clark and Tilman, 2008; Zhang et al., 2014; van den Berg et al., 2016). However, there is limited literature documenting the effects of N deposition on functional traits, except for a few scholars who highlighted the application of trait-based approaches to exploring the response of plants to N deposition (De Deyn et al., 2008; Lavorel et al., 2013). Functional-based hypotheses insisted that plant interactions will shift from soil N competition to light competition (Suding et al., 2005; Stevens et al., 2006; Hautier et al., 2009; Bobbink et al., 2010; Cleland and Harpole, 2010; Borer et al., 2014; Humbert et al., 2016; Ma et al., 2019) with the increase in the N deposition rate, potentially affecting the functional traits of plants related to light acquisition or N availability, such as height or specific leaf area (SLA) (Reich et al., 1998; Diekmann and Falkengren-Grerup, 2002). N deposition can positively affect the functional groups of grass by promoting their growth and negatively impacting other functional groups, such as forbs by limiting their growth (Johansson et al., 2012; Borer et al., 2014; Humbert et al., 2016; Zhang et al., 2020). Until present, the limited studies from temperate and tropical forests have shown inconsistent results about the responses of the functional traits of plants to N deposition, i.e., some scholars stated that functional traits were the conductors of the N deposition on communities (Wang et al., 2018), whereas some other scholars reported that plant traits were not related to N deposition (Moreau et al., 2015). In addition, it is even less clear about the responses of functional traits of plants from dominant species to functional groups, and whole communities in different types of grassland to N deposition were consistent with the same or different.

As for alpine grassland ecosystems on the QTP, few researchers other than Wang et al. (2018) have explored the effects of N addition on the functional traits of plants of the alpine meadow. AS is one of the most important ecosystems on the QTP, and cultivated grassland (CG) is the key intervention to prevent severe grassland degradation driven mainly by climatic change and livestock overgrazing (Zhao et al., 2017; Zhang et al., 2020); both of them are critically important for sustainable development of both upstream and downstream regions. On the one hand, according to the mass ratio hypothesis, the ecosystems are largely controlled by the characteristics of dominant species or the dominant functional group (Grime, 1998); in contrast, the complementary effect may be important for the responses to N deposition, as the responses may be eliminated or weakened by complex species interactions (Cleland et al., 2006). Therefore, studies are needed to clarify the impacts of acidification, eutrophication, and P limitation associated with N deposition on the functional traits of plants from dominant species to functional groups and communities in AS and CG ecosystems. Within this context, we conducted the manipulated experiments with N addition at different gradients in both AS and CG on the QTP to test three hypotheses, namely, (1) eutrophication, acidification, and P availability associated with N deposition impact the functional traits of plants of alpine grasslands; (2) there are cascading effects of N deposition on the functional traits of plants from dominant species to functional groups and whole communities of the alpine grasslands; and (3) the responses of alpine plant functional traits to N deposition vary with grassland types.

## Materials and Methods

### Study Site

**FIGURE 1:**
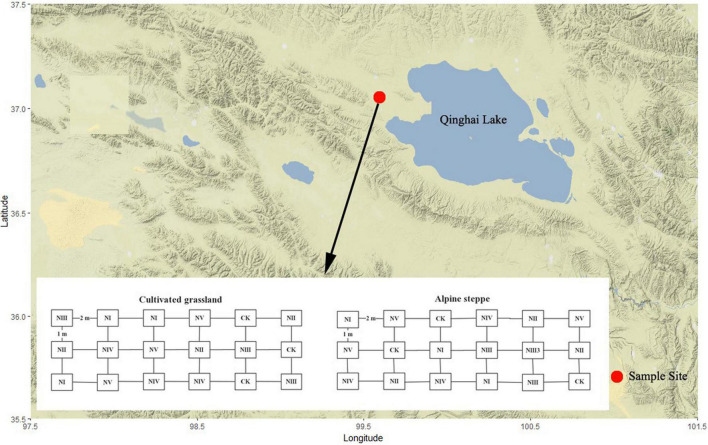
Geographical location of the study site on the terrain-background map. CK, no N addition, NI, addition at 8 kg N/ha/year, NII, addition at 24 kg N/ha/year, NIII, addition at 40 kg N/ha/year, NIV, addition at 56 kg N/ha/year, and NV, addition at 72 kg N/ha/year.

This study was conducted in Tiebujia town, Gonghe County (99°35′N, 37°02′E, 3,270 m ASL), which is located at the west side of Qinghai Lake (Figure 1) in Qinghai Province, China. In this town, the mean annual temperature is approximately −0.4 to 1.2°C, the mean annual precipitation is approximately 360–430 mm, and the annual evaporation capacity is around 1,550 mm. The typical vegetation is AS, dominated by *Stipa capillata*. The CG next to the AS was plowed before and planted with *Elymus brevaristatus* in 2012. In both grasslands, the soil is predominantly sandy loam.

### Experimental Design

In May 2015, 18 plots (i.e., replicates) of 2 m × 5 m were, respectively, placed in uniform sites (with similar topography, vegetation, and soil) of AS and CG. The plots were randomly treated by six levels of N addition with granular ammonium nitrate (NH_4_NO_3_) mixed with soil in three replicates for each N treatment, 0 N (CK), 8 kg N/ha/year (NI), which equals to the annual N deposition on the QTP reported by Lu and Tian in 2007 (Lu and Tian, 2007), 24 kg N/ha/year (NII), 40 kg N/ha/year (NIII), 56 kg N/ha/year (NIV), and 72 kg N/ha/year (NV) (Figure 1), and we applied N monthly basis in the growing season. We recorded the species composition of 72 sampling plots (6 treatments × 3 replicates × 2 grassland types × 2 years) at the peak growth rate in late July to early August of 2015 and 2016.

### Soil Sampling

We sampled three cores of the surface soil (0–20 cm and 3.5 cm in diameter) from each sampling plot and mixed them well in the mid-August of 2015 and 2016. The soil samples were sealed in polyethylene bags and transported to the lab for extraction after being air-dried, ground, and sieved (18 mesh sieves were used for pH measurement, and 100 mesh sieves were used for soil nutrient measurement). We used a glass electrode to measure the soil pH in the supernatant by homogeneously mixing 5 g of soil and 25 ml of water. Total nitrogen (TN) was measured using an element analyzer (EA 3000, Italy). NH_4_-N and NO_3_-N were measured using a flow injection autoanalyzer (AACE, Germany). The concentrations of total phosphorus (TP) and available phosphorus (AP) were measured using inductively coupled plasma spectrometers (ICP; SPECTRO ARCOS EOP, Germany). The measurement of soil nutrients is performed following the approach of Han et al. (2019).

### Plant Sampling

We randomly placed one 1 m × 1 m quadrat for sampling plant species composition of 72 sampling plots (6 treatments × 3 replicates × 2 grassland types × 2 years) at the peak growth rate in late July to early August of 2015 and 2016; the survey included species name, abundances for each species, plant height, and plant coverage (visual estimation of % cover of each species). We recorded 28 species in AS, including 9 species of non-forb and 19 species of forb, and 25 species in CG, including 9 species of non-forb and 16 species of forb. We randomly selected more than five individuals of each species in each plot for measuring the plant height (CI-202, America); plant height was the basic and easily measurable functional trait, and it significantly and positively correlated with N deposition (Diekmann and Falkengren-Grerup, 2002). In addition, we also calculated the SLA (i.e., a measure of projected leaf area per unit dry mass) by measuring leaf area (CI-202, America) and leaf weight (i.e., balance); the SLA was related to the changing pattern of the photosynthesis-N relationship based on mass and area (Reich et al., 1998). At the species level, we chose the dominant species (i.e., *Stipa capillata* in AS and *Elymus breviaristatus* in CG), since both coverages of *Stipa capillata* (about 15%) and *Elymus breviaristatus* (about 35%) in each plot were the largest in all species; at the functional group level, we divided different species into forb and non-forb, since there were few species for sedge and legume; at the community level, we calculated the community-weighted mean value (CWM) (Lavorel et al., 2008) by combining species relative abundances with population-based plant height and plant SLA from each plot.

### Statistical Analysis

We modeled plant height, SLA, CWM height, and CWM-SLA of AS and CG as dependent variables, and pH, TN, NH4-N, NO3-N, AP, and TP as independent variables using the linear mixed-effects model (LMM). In the models, replicates were included as a random effect to account for spatial autocorrelation, and years were included as a random effect to annual variations in functional traits at the species level and CWMs within the same grassland type and treatment; replicates, years, and species (i.e., variations among different species of the same functional group) were included as a random effect for the functional group level within the same grassland type and treatment.

To assess the relative influence of N addition gradients, acidification, eutrophication, and P limitation on functional traits and CWMs of different grassland types, we tested eight candidate models, namely, (1) a null model with only log_10_ N addition gradients (Zhang et al., 2016), i.e., considering background value reported by Lu and Tian in 2007 (Lu and Tian, 2007), of 8 kg N/ha/year of N deposition in the Qinghai-Tibet Plateau, 8 kg N/ha/year for CK, 16 kg N/ha/year for NI, 30 kg N/ha/year for NII, 48 kg N/ha/year for NIII, 64 kg N/ha/year for NIV, 80 kg N/ha/year for NV, and random effects for both AS and CG; (2) a eutrophication model in which TN and NH_4_-N were added to the null model for AS, and TN, NH_4_-N, and NO_3_-N were added to the null model for CG; (3) an acidification model in which pH was added to the null model for both AS and CG; (4) a P availability model in which AP and TP were added to the null model for AS, and TP was added to the null model for CG; (5) a eutrophication and acidification model in which TN, NH_4_-N, and pH were added to the null model for AS, and TN, NH_4_-N, NO_3_-N, and pH were for CG; (6) a eutrophication and P availability model in which TN, NH_4_-N, AP, and TP were added to the null model for AS, and TN, NH_4_-N, NO_3_-N, and TP for CG; (7) an acidification and P availability model in which pH, AP, and TP were added to the null model for AS, and pH and TP for CG; (8) a full model including all variables (i.e., log_10_ N addition gradients, TN, NH_4_-N, pH, AP, and TP) for AS, and log_10_ N addition gradients, TN, NH_4_-N, pH, and TP for CG. There was collinearity between NO_3_-N and NH_4_-N (Pearson correlation coefficient = −0.86), and NH_4_-N and TP (Pearson correlation coefficient = 0.70) in AS (Supplementary Figure 1); AP and TP (Pearson correlation coefficient = 0.76), and NH_4_-N and AP (Pearson correlation coefficient = −0.77) in CG (Supplementary Figure 2). The best model was chosen by the lowest Akaike Information Criterion (AIC) models (Burnham and Anderson, 2002), within 2 units are generally considered to have equal weight (Lin et al., 2014), and the predictor variables of the best model were evaluated with the largest statistically significant parameter values (based on standardized data), judged as having the greatest relative impact on functional traits and CWMs of different grassland types, and marginal R^2^ represents the variance explained by fixed factors, and conditional R^2^ is interpreted as variance explained by both fixed and random factors (Johnson, 2014).

We used the “FD” packages to calculate CWM; “lme4” and “lmerTest” packages to perform LMM, and “MuMIn” packages to perform the goodness of fit (pseudo-R^2^) of the LMM (Zurr et al., 2009) in the R version 3.5.1 (R Development Core Team).

## Results

### Responses of Plant Functional Traits at the Species Level

**FIGURE 2:**
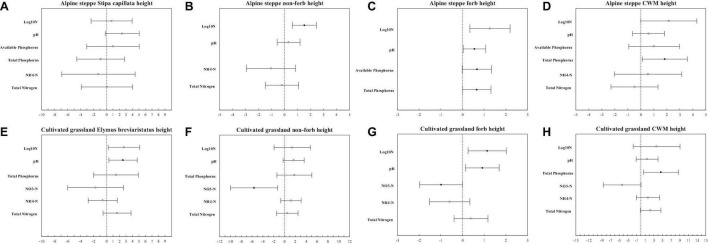
Standardized parameter estimates (±2 SE) of log_10_ nitrogen addition gradients, pH, available phosphorus, total phosphorus NH_4_-N, NO_3_-N, and total nitrogen for *Stipa capillata* height **(A)**, non-forb height of alpine steppe **(B)**, forb height of alpine steppe **(C)**, community weighted mean value of height of alpine steppe **(D)**, *Elymus breviaristatus* height **(E)**, non-forb height of cultivated grassland **(F)**, forb height of cultivated grassland **(G)**, and community-weighted mean value of height of cultivated grassland **(H)**. Filled circles indicate significant effects (*p* < 0.05).

**FIGURE 3:**
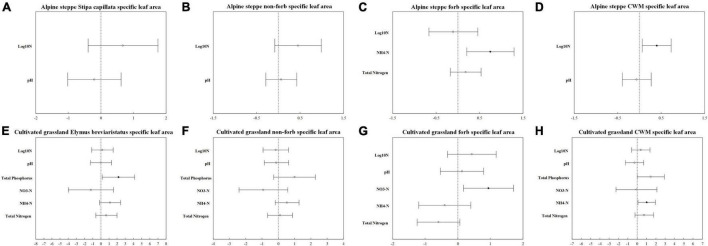
Standardized parameter estimates (±2 SE) of log_10_ nitrogen addition gradients, pH, available phosphorus, total phosphorus NH_4_-N, NO_3_-N, and total nitrogen for *Stipa capillata* SLA **(A)**, non-forb SLA of alpine steppe **(B)**, forb SLA of alpine steppe **(C)**, community-weighted mean value of SLA of alpine steppe **(D)**, *Elymus breviaristatus* SLA **(E)**, non-forb SLA of cultivated grassland **(F)**, forb SLA of cultivated grassland **(G)**, and community-weighted mean value of SLA of cultivated grassland **(H)**. Filled circles indicate significant effects (*p* < 0.05).

The model with all factors at the species level provided the best fit for the height of *Stipa capillata* and *Elymus breviaristatus*, the SLA of *Elymus breviaristatus* with the lowest AIC values (Tables 1, 2), indicating that N addition gradients, acidification, eutrophication, and P availability showed significant effects on *Stipa capillata* height (Figure 2A), *Elymus breviaristatus* height, and *Elymus breviaristatus* SLA. Soil pH was the most important predictor of *Elymus breviaristatus* height, and the effect was positive (Figure 2E), but for *Elymus breviaristatus*, SLA and TP were the most important predictors (Figure 3E).

**TABLE 1:** ΔAIC values of the linear mixed models of plant height and community weighted mean value of height in alpine steppe and cultivated grassland.

	Alpine steppe				Cultivated grassland			
	Species	Non-forb	Forb	Community	Species	Non-forb	Forb	Community
(1)	16.35	4.01	8.46	11.93	16.63	18.75	12.56	24.87
(2)	9.60	2.55	10.22	9.08	11.32	7.96	6.42	12.75
(3)	9.80	3.21	3.12	7.70	11.43	15.54	8.45	18.22
(4)	14.02	5.09	4.67	8.82	16.98	17.75	13.89	19.42
(5)	3.21	**0**	6.12	4.95	2.46	3.95	**0**	6.75
(6)	6.72	3.01	8.89	3.59	7.95	6.45	7.12	5.42
(7)	6.82	2.71	**0**	5.32	10.31	13.54	9.75	5.35
(8)	**0**	**0.58**	3.98	**0**	**0**	**0**	**1.45**	**0**

*AIC values of the best models are shown in bold. (1) represents only log_10_ N addition gradients; (2) represents N addition gradients and eutrophication; (3) represents N addition gradients and acidification; (4) represents N addition gradients and P limit; (5) represents N addition gradients, eutrophication, and acidification; (6) represents N addition gradients, eutrophication, and P limit; (7) represents N addition gradients, acidification, and P limit; and (8) represents N addition gradients, acidification, eutrophication, and P limit.*

**TABLE 2:** ΔAIC values of the linear mixed models of SLA and community weighted mean value of SLA of alpine steppe and cultivated grassland.

	Alpine steppe				Cultivated grassland			
	Species	Non-forb	Forb	Community	Species	Non-forb	Forb	Community
(1)	3.04	**0.52**	3.31	**1.89**	8.88	4.14	8.46	7.67
(2)	2.52	2.61	**0**	2.23	3.06	**1.24**	**1.02**	2.25
(3)	**0**	**0**	3.45	**0**	5.42	2.54	7.43	5.29
(4)	6.39	6.60	9.42	7.43	16.07	4.85	10.32	15.80
(5)	**0.86**	2.01	**0.30**	**1.65**	7.84	**0.15**	**0**	8.27
(6)	4.02	6.23	5.48	4.61	10.79	**1.02**	2.54	10.54
(7)	4.02	5.96	9.75	7.26	12.97	3.43	9.33	13.38
(8)	**1.59**	5.62	6.21	4.33	**0**	**0**	**1.50**	**0**

*AIC values of the best models are shown in bold. (1) represents only log_10_ N addition gradients; (2) represents N addition gradients and eutrophication; (3) represents N addition gradients and acidification; (4) represents N addition gradients and P limit; (5) represents N addition gradients, eutrophication, and acidification; (6) represents N addition gradients, eutrophication, and P limit; (7) represents N addition gradients, acidification, and P limit; and (8) represents N addition gradients, acidification, eutrophication, and P limit.*

The model with log_10_ N addition gradients and pH provided the best fit for *Stipa capillata* SLA (Table 2), indicating that N addition gradients and acidification presented significant effects on *Stipa capillata* SLA (Figure 3A). However, low values of R^2^ (0.09) of fixed factors suggested that much of the variation in *Stipa capillata* SLA remained unexplained (Table 3).

**TABLE 3:** The pseudo-R^2^ of the linear mixed models of plant height and community weighted mean value of height in alpine steppe and cultivated grassland.

Rm^2^/Rc^2^	Alpine steppe				Cultivated grassland			
	Species	Non-forb	Forb	Community	Species	Non-forb	Forb	Community
(1)	0.01/0.09	0.02/0.61	0.05/0.67	0.23/0.46	0.18/0.21	0.02/0.78	0.03/0.66	0.14/0.44
(2)	0.14/0.14	0.04/0.65	0.05/0.70	0.23/0.81	0.22/0.24	0.05/0.81	0.09/0.66	0.21/0.54
(3)	0.07/0.24	0.02/0.61	0.06/0.69	0.25/0.49	0.26/0.35	0.03/0.78	0.04/0.66	0.19/0.45
(4)	0.03/0.12	0.02/0.62	0.09/0.68	0.32/0.53	0.21/0.22	0.03/0.79	0.04/0.65	0.21/0.51
(5)	0.22/0.22	**0.04/0.64**	0.06/0.70	0.22/0.78	0.28/0.64	0.07/0.83	**0.11/0.68**	0.26/0.54
(6)	0.14/0.14	0.03/0.62	0.09/0.68	0.37/0.51	0.25/0.27	0.10/0.86	0.10/0.67	0.30/0.62
(7)	0.09/0.28	0.02/0.62	**0.10/0.70**	0.33/0.56	0.28/0.28	0.04/0.80	0.09/0.66	0.23/0.50
(8)	**0.22/0.22**	**0.03/0.62**	0.10/0.70	**0.37/0.53**	**0.27/0.52**	**0.15/0.90**	**0.11/0.68**	**0.31/0.63**

*Rm^2^ represents the variance explained by fixed factors; Rc^2^ is interpreted as variance explained by both fixed and random factors. (1) represents only log_10_ N addition gradients; (2) represents N addition gradients and eutrophication; (3) represents N addition gradients and acidification; (4) represents N addition gradients and P limit; (5) represents N addition gradients, eutrophication, and acidification; (6) represents N addition gradients, eutrophication and P limit; (7) represents N addition gradients, acidification, and P limit; and (8) represents N addition gradients, acidification, eutrophication, and P limit. AIC values of the best models are shown in bold.*

### Responses of Plant Functional Traits at the Functional Group Level

The model with all factors for the functional groups of non-forbs and forbs in the CG provided the best fit for both height and SLA with the lowest AIC values (Tables 1, 2), indicating that both N addition gradients, acidification, eutrophication, and P availability showed significant effects on the height and SLA of non-forbs and forbs in the CG. However, low values of R^2^ (0.05) of fixed factors for non-forb SLA (Figure 3F) in the CG suggested that much of the variation in non-forb SLA remained unexplained (Table 4). NH_4_-N was the most important predictor with negative impacts for the height of non-forb (Figure 2B) and forb functional groups in the CG (Figures 2F,G). In addition, log_10_ N and pH were the most important predictors with positive impacts for the height of forb functional groups in the CG (Figure 2G). NO_3_-N was the most important predictor with a positive impact for the SLA of the forb group (Figure 3G).

**TABLE 4:** The pseudo-R^2^ of the linear mixed models of specific leaf area (SLA) and community weighted mean value of SLA of alpine steppe and cultivated grassland.

Rm^2^/Rc^2^	Alpine steppe				Cultivated grassland			
	Species	Non-forb	Forb	Community	Species	Non-forb	Forb	Community
(1)	0.08/0.24	0.03/0.21	>0.01/0.37	**0.16/0.16**	>0.01/0.02	>0.01/0.33	0.03/0.66	>0.01/0.16
(2)	0.10/0.26	0.04/0.30	**0.04/0.41**	0.34/0.83	0.10/0.10	0.07/0.32	**0.09/0.66**	0.09/0.26
(3)	**0.09/0.22**	**0.03/0.21**	>0.01/0.37	**0.16/0.16**	0.03/0.03	>0.01/0.32	0.04/0.66	0.01/0.13
(4)	0.09/0.24	0.04/0.26	0.01/0.36	0.19/0.19	0.16/0.60	0.05/0.53	0.04/0.65	0.14/0.69
(5)	0.11/0.23	0.04/0.29	0.04/0.41	**0.34/0.84**	0.11/0.11	**0.07/0.32**	**0.11/0.68**	0.12/0.21
(6)	0.13/0.25	0.11/0.51	0.04/0.41	0.34/0.93	0.19/0.66	0.05/0.43	0.10/0.67	0.20/0.84
(7)	0.09/0.24	0.04/0.26	>0.01/0.36	0.19/0.19	0.15/0.57	0.06/0.55	0.09/0.66	0.14/0.71
(8)	0.14/0.23	0.11/0.50	0.04/0.41	0.34/0.93	**0.18/0.67**	**0.05/0.49**	**0.11/0.68**	**0.21/0.87**

*Rm^2^ represents the variance explained by fixed factors; Rc^2^ is interpreted as variance explained by both fixed and random factors. (1) represents only log_10_ N addition gradients; (2) represents N addition gradients and eutrophication; (3) represents N addition gradients and acidification; (4) represents N addition gradients and P limit; (5) represents N addition gradients, eutrophication, and acidification; (6) represents N addition gradients, eutrophication, and P limit; (7) represents N addition gradients, acidification, and P limit; and (8) represents N addition gradients, acidification, eutrophication, and P limit. AIC values of the best models are shown in bold.*

The best model with the lowest AIC values for the height and SLA of non-forbs and forbs in the AS was inconsistent, e.g., N addition gradients and acidification were found as the key impact factors for the height of the non-forb functional group in the AS (Table 1), while the N addition gradients, acidification, and P availability were found as the key impact factors for the height of the forb functional group in the AS (Table 1). N addition gradients and acidification were identified as the key impact factors for the SLA of the non-forb functional group (Figure 3B) in the AS (Table 2), whereas N addition gradients and eutrophication were identified as the key impact factors for the SLA of the forb functional group in the AS (Table 2). However, values of R^2^ of fixed factors were low (< 0.10) for non-forb height in the AS, non-forb SLA in the AS, and forb SLA in the AS (Tables 3, 4). AP, TP, and pH were the most important predictors with positive effects for the height of the forb functional group in the AS (Figure 2C). Ammoniacal N was the most important predictor with positive effects for the SLA of the forb functional group in the AS (Figure 3C).

### Responses of Plant Functional Traits at the Community Level

The model with all factors at CWM height in AS and CG, CWM SLA in CG, provided the best fit with the lowest AIC values (Tables 1, 2), indicating that N addition gradients, acidification, eutrophication, and P availability showed significant effects on the CWM height of plant communities in the AS and CG and on the CWM SLA in the CG (Figures 3D,H). TP was the most important predictor with positive effects for CWM height of plant communities in AS and CG (Figures 2D,H).

The model with log_10_ N addition gradients and pH at CWM SLA in AS provided the best fit with the lowest AIC values (Table 2), indicating that N addition gradients and acidification showed significant effects on CWM SLA of plant communities in the AS. The N addition gradient was the most important predictor with positive effects for CWM SLA of plant communities in the AS (Figure 3D).

## Discussion

At the species level, the responses of the height of *Stipa capillata* and the height and SLA of *Elymus breviaristatus* to N deposition can support our first hypothesis, i.e., eutrophication, acidification, and P availability associated with N deposition interdependently impact the functional traits of plants of alpine grasslands. This is consistent with viewpoints of previous researchers that N, P, and pH were strongly interdependent with plant metabolism (Stevens et al., 2010; Phoenix et al., 2012; Laliberté et al., 2014). However, we observed that the SLA of *Stipa capillata* did not support this hypothesis, i.e., only acidification associated with N addition gradients was related to the SLA of *Stipa capillata*. In this study, we found that the SLA of *Stipa capillata* was not significantly correlated with any soil factors. We speculated that this may be related to the leaf configuration and leaf structure of *Stipa* spp., whose leaf blade is naturally curled and needle-shaped, unlike *Elymus breviaristatus*. Besides, the result from this study was inconsistent with the previous finding that plant height was significantly positively correlated with N deposition (Diekmann and Falkengren-Grerup, 2002). This could be buffer feedback for the alpine grassland ecosystem in coping with N deposition.

At the functional group level, non-forb functional groups were higher and larger than the forb functional groups due to stronger light competition under N deposition (Suding et al., 2005; Stevens et al., 2006; Cleland and Harpole, 2010; Borer et al., 2014), meaning that acidification, eutrophication, and P availability associated with N deposition were more conducive to non-forb functional group species. We observed that in log_10_ N addition gradients, NO_3_-N was significantly positively related to plant height of non-forb functional groups in the AS; pH, AP, and TP were also significantly related to the plant height of forb functional groups in the AS. In contrast, we found that NO_3_-N was significantly negatively related to the plant height of both forb and non-forb functional groups in the CG. This can support our third hypothesis, i.e., the responses of alpine plant functional traits to N deposition vary with grassland types/land-use types. The different responses between non-forb functional groups and forb functional groups in the same type of alpine grasslands to N application may be related to competitive interaction (Kardol et al., 2010); the subdominant species (e.g., forb species in either AS or CG) may respond to the changed competitive interaction with the dominant species (e.g., non-forb species in either AS or CG). In addition, we found that the SLA of forb functional groups was more sensitive to soil factors than the SLA of non-forb functional groups in both AS and CG. We speculated that the responses of the non-forb functional group may be light-driven, shifting from soil N competition to light competition (Stevens et al., 2006; Cleland and Harpole, 2010; Borer et al., 2014; Zhang et al., 2020), while the responses of the forb functional groups may be nutrient-driven, competing for nutrients especially in the context of N deposition. Further studies are needed to test this assumption.

At the community level, we confirmed our first hypothesis by finding that the eutrophication, acidification, and P availability associated with N deposition inter- impacted the CWM height of plant communities in both AS and CG and the CWM SLA of the plant community in the CG. Since the abundance of each species was considered in the analysis, CWMs of plant communities would significantly increase due to the niche complementarity or over-yielding in the context of N addition (Wang et al., 2018), mainly due to increased dominance of grasses and the decreased dominance of forbs (Zavaleta et al., 2003; Clark and Tilman, 2008; Zhang et al., 2014, 2020; van den Berg et al., 2016). However, we only observed a similar phenomenon on the SLA of plant communities in the AS. We assumed the P availability associated with N deposition may play determining roles for this, as we found that TP and AP were significantly related to the CWM height of plant communities in the AS and the CGs and CWM SLA of the plant community in the CG. Although our previous studies indicated that lower than 14 of soil N:P ratio in both AS and CG along low-high N addition gradients (Han et al., 2019) implied these alpine grasslands were ecosystems with N limitation rather than P limitation (Stevens et al., 2006), the results derived from the responses of the functional traits of plants in this study suggest that it might be biased to conclude the ecosystems are N limited or P limited solely based on the soil N:P ratio.

The responses of the functional traits of alpine plants to N depositions showed the cascading effects from dominant species to functional groups and plant communities. This supports our second hypothesis. We assumed that the synchronous or asynchronous reactions of alpine plant traits of dominant species to functional groups and plant communities to N depositions were dependent mainly on two effects, namely, the mass effect and complementary effect. The mass effect hypothesis (Grime, 1998) emphasized that the ecosystems are largely controlled by the characteristics of dominant species or functional groups. The complementary effect may be important for the responses to N deposition, as the responses may be eliminated or weakened by complex species interactions (Cleland et al., 2006). We believed that the cascading responses from dominant grass species to non-forb functional groups were mainly controlled by mass effects, and the cascading responses from functional groups and plant communities were mainly regulated by the complementary effect, since the different reactions between non-forb plants and forb plants to N deposition may modify the overall biotic responses at the community level. The different cascading reactions of plant functional traits from dominant species to functional groups and plant communities to N deposition imply that the alpine grassland ecosystem is sensitive to N deposition. This is inconsistent with the finding of Wang et al. (2018) from the alpine meadow, which stressed that plant community-level traits can mediate effects of N addition. We think it is necessary to deduce the cascading responses of alpine plants to N deposition by combining the results from different alpine ecosystems.

Although we found that most of the cascading effects were synchronous in both the AS and the CG, we did catch a signal of the “insurance effect” in the SLA of forbs in the AS but not in the CG. As species diversity affected the stability of resistance of ecosystem functions to environmental disturbances according to the “insurance hypothesis” (Kahmen et al., 2005), we did see more resistance of plant functional traits, such as the heights and SLA in the AS with higher species diversity than the CG with lower species diversity in response to increasing N deposition. This can further support our third hypothesis that grassland types/land-use types can alter the responses of alpine plant functional traits to N deposition.

In summary, we applied eight linear mixed-effect models to explore the synchronous or asynchronous responses of cascading effects of plant functional traits (particularly, plant height and SLA) from dominant species to functional groups and whole communities in different types of grassland to N deposition. In conclusion, most of the responses of plant functional traits to acidification, eutrophication, and P availability associated with N deposition in the AS and the CG were synchronous, while only the response of the SLA of forb functional groups to eutrophication associated with N deposition in the AS was asynchronous. These findings may provide new insights for facilitating sound adaption strategies for sustainable grassland management to cope with N deposition in the alpine regions of the QTP or worldwide.

## Data Availability Statement

The raw data supporting the conclusions of this article will be made available by the authors, without undue reservation.

## Author Contributions

SL: investigation, data curation, formal analysis, and writing the original draft. ZZ: conceptualization and review. SD: funding acquisition, investigation, project administration, resources, supervision, and writing review and editing. HS, YX, JX, XG, SW, and PS: investigation and data curation. All authors contributed to the article and approved the submitted version.

## Conflict of Interest

The authors declare that the research was conducted in the absence of any commercial or financial relationships that could be construed as a potential conflict of interest.

## Publisher’s Note

All claims expressed in this article are solely those of the authors and do not necessarily represent those of their affiliated organizations, or those of the publisher, the editors and the reviewers. Any product that may be evaluated in this article, or claim that may be made by its manufacturer, is not guaranteed or endorsed by the publisher.
