# Supplementary material for: Synchronous Responses of Plant Functional Traits to Nitrogen Deposition From Dominant Species to Functional Groups and Whole Communities in Alpine Grasslands on the Qinghai-Tibetan Plateau
Source: Front Plant Sci. 2022 Feb 9;13:827035. doi: 10.3389/fpls.2022.827035 (PMC8864091; doi:10.3389/fpls.2022.827035)
Supplement: Supplementary file 1 [file Data_Sheet_1.docx]

Fig S1. Pearson correlation coefficients of log_10_ nitrogen addition gradients and soil factors in the alpine steppe, ^*^ *p* < 0.05, ^**^ *p* < 0.01, ^***^ *p* < 0.001.

Fig S2. Pearson correlation coefficients of log_10_ nitrogen addition gradients and soil factors in the cultivated grassland, ^*^ *p* < 0.05, ^**^ *p* < 0.01, ^***^ *p* < 0.001.

**Table** S**1** The relationships between functional traits and soil factors

| Grassland types | | functional traits | log_10_ N | pH | Total Nitrogen | NO_3_-N | NH_4_-N | Total Phosphorus | Available Phosphorus |
| --- | --- | --- | --- | --- | --- | --- | --- | --- | --- |
| Alpine steppe | height | species | 0.11 | 0.17 | 0.20 | -0.37* | 0.28 | -0.25 | -0.10 |
|  |  | non-Forb | 0.18** | -0.05 | 0.12 | -0.16* | 0.11 | -0.05 | 0.04 |
|  |  | forb | 0.24*** | 0.04 | -0.05 | 0.20** | -0.18** | 0.19** | 0.27*** |
|  |  | community | 0.50** | 0.01 | -0.03 | 0.06 | -0.05 | 0.26 | 0.43** |
|  | specific leaf area | species | 0.30 | -0.19 | 0.01 | 0.13 | -0.08 | -0.10 | 0.11 |
|  |  | non-Forb | 0.16* | -0.05 | 0.08 | -0.05 | 0.07 | -0.06 | 0.01 |
|  |  | forb | -0.02 | -0.01 | 0.14* | -0.07 | 0.16* | -0.03 | -0.10 |
|  |  | community | 0.40* | -0.14 | 0.26 | -0.21 | 0.31 | -0.21 | -0.02 |
| Cultivated grassland | height | species | 0.44** | 0.36* | 0.28 | 0.04 | 0.05 | -0.06 | -0.07 |
|  |  | non-Forb | 0.30** | 0.07 | 0.19 | 0.14 | 0.14 | -0.19 | -0.20 |
|  |  | forb | 0.24** | 0.22** | 0.08 | -0.34*** | -0.14 | 0.24* | 0.34*** |
|  |  | community | 0.38* | 0.35* | 0.27 | -0.19 | 0.08 | 0.21 | 0.18 |
|  | specific leaf area | species | -0.03 | 0.16 | 0.04 | -0.17 | 0.17 | 0.21 | 0.05 |
|  |  | non-Forb | -0.06 | 0.07 | -0.05 | -0.19 | 0.02 | 0.16 | 0.12 |
|  |  | forb | 0.03 | 0.03 | -0.19* | 0.22* | 0.07 | -0.01 | -0.09 |
|  |  | community | 0.01 | 0.18 | 0.03 | -0.28 | 0.17 | 0.26 | 0.18 |

^*^ *p* < 0.05, ^**^ *p* < 0.01, ^***^ *p* < 0.001, log_10_N represents log10 nitrogen addition gradients.
